# Systematic review and meta-analysis of docetaxel perioperative chemotherapy regimens in gastric and esophagogastric tumors

**DOI:** 10.1038/s41598-019-52334-y

**Published:** 2019-11-01

**Authors:** Pedro Luiz Serrano Uson Junior, Vanessa Montes Santos, Diogo Diniz Gomes Bugano, Elivane da Silva Victor, Edna Terezinha Rother, Fernando Cotait Maluf

**Affiliations:** Hospital Israelita Albert Einstein, Oncology Department, 627/701 Avenida Albert Einstein, Morumbi, São Paulo Brazil

**Keywords:** Chemotherapy, Gastric cancer

## Abstract

FLOT regimen became the standard perioperative treatment in several centers around the world for esophagogastric tumors despite concerns about toxicity. In addition, FLOT has never been compared with other docetaxel-based regimens. To address this question, we conducted a systematic review of PubMed, Embase and Web of Science including prospective or retrospective studies of docetaxel based perioperative regimen in gastric and esophagogastric tumors. Data regarding chemotherapy regimens, efficacy and toxicity were extracted. Outcomes were compared using a random effects model. Of 548 abstracts, 16 were considered eligible. Comparing the studies with meta-analysis we can see that the regimens are similar in terms of pathological complete response, resection rate, progression free survival and overall survival in one year, without significant heterogeneity. The meta-regression of docetaxel dose failed to show any association with dose ranging between 120–450 mg/m². Regarding the toxicity of the regimens it is noted that the regimens are quite toxic (up to 50–70% of grade 3–4 neutropenia). The results of this meta-analysis with a combined sample size of more than 1,000 patients suggest that docetaxel perioperative regimens are equivalent in outcomes. Prospective trials addressing modified regimens should be performed to provide less toxic strategies and be applicable to all patients.

## Introduction

Gastric cancer is the fifth leading cause of cancer in the world population and geographically more frequent in Asian, Eastern European and South American countries.^1^ Gastric cancer represented up to 28,000 of all new cancer cases in United States last year^2^. The mortality rate of gastric cancer has decreased through last decades mainly because of changes of lifestyle, control of Helicobacter pylori infection and advances in diagnosis and treatment methods^3,4^.

The main treatment of non-metastatic gastric cancer is surgical resection associated with perioperative chemotherapy. Recently, a phase 3 randomized trial showed improved overall survival among patients with operable gastric cancer treated with perioperative chemotherapy based on docetaxel, oxaliplatin and 5FU (FLOT) over epirubicin, cisplatin, and fluorouracil or capecitabine (ECF/ECX)^5,6^.

FLOT is the best regimen of docetaxel-based perioperative chemotherapy. The evaluation of the eligibility of this treatment for patients with poor performance or for elderly and the toxicity profile is a subject under discussion^7,8^.

Other docetaxel-based regimens in the perioperative setting have been previously studied and apparently, they present similar results to the FLOT regimen with different toxicity profiles, however, direct comparisons between these different regimens have not been performed^9–11^.

This study is a systematic review and meta-analysis of prior publications evaluating different docetaxel based on perioperative chemotherapy regimens regarding efficacy endpoints including complete response and resection rates, progression free and overall survival. Furthermore, protocols will be evaluated regarding toxicity of the regimens.

## Materials and Methods

### Search strategy

**Figure 1:**
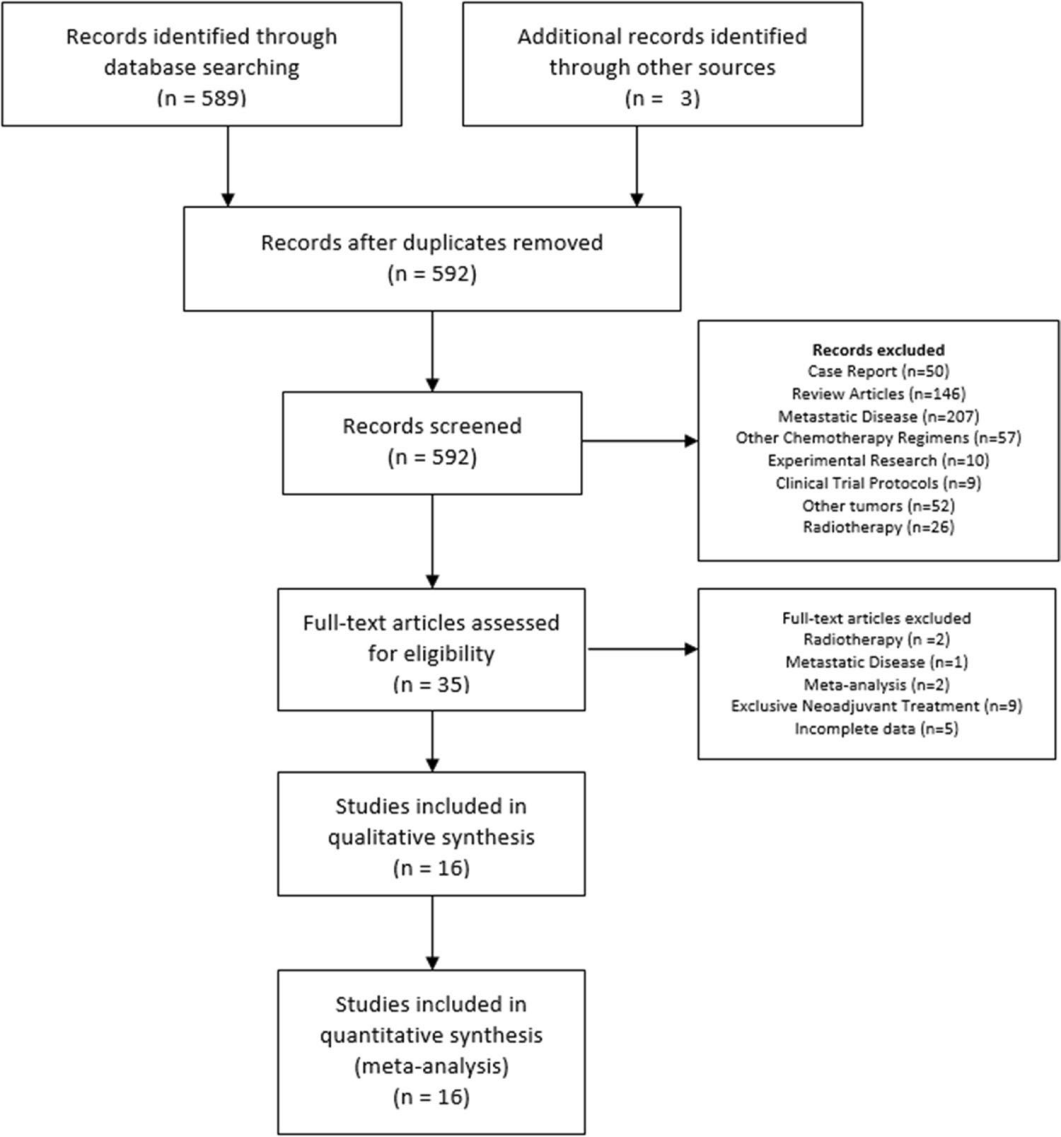
Flow Diagram of included studies.

This study was designed in conformity with the guidelines of the 2009 Preferred Reporting Items for Systematic Reviews and Meta-Analysis (PRISMA) statement^12^. We searched PubMed (1950–2019) on May 2019. We used the terms ((((((esophagogastric junction[Title/Abstract]) OR ((esophagus neoplasms[Title/Abstract]) OR esophageal neoplasms[Title/Abstract])) OR ((stomach neoplasms[Title/Abstract]) OR gastric neoplasms[Title/Abstract]))) AND (((FLOT[Title/Abstract]) OR “DCF”[Title/Abstract])))) OR (((((esophagogastric junction[Title/Abstract]) OR ((esophagus neoplasms[Title/Abstract]) OR esophageal neoplasms[Title/Abstract])) OR ((stomach neoplasms[Title/Abstract]) OR gastric neoplasms[Title/Abstract]))) AND ((docetaxel[Title/Abstract]) OR Antineoplastic Combined Chemotherapy Protocols[Text Word])) with 169 abstracts. We searched Embase with the same terms yielding more 296 abstracts, and Web of Science with more 138 abstracts. After removing duplicates and adding 3 more records identified through other sources, the final database had 592 records (Fig. 1). The protocol was registered in system research project manager (SGPP) of Albert Einstein Hospital and this is available for consultation on request (number 3494-18).

Two authors (P.L.S.U.J. and V.M.S.) reviewed all abstracts. Inclusion criteria were: (1) prospective or retrospective cohorts; (2) patients with gastric and/or esophagogastric junction adenocarcinoma; (3) use of docetaxel based perioperative chemotherapy regimen; (4) available information of at least one efficacy endpoint (complete response rate, resection rate, progression free survival or overall survival); Exclusion criteria were: (1) language other than English; (2) inability to discern metastatic patients from locally advanced patients, and studies with only metastatic patients; (3) duplicate publication; (4) review articles and case reports; (5) other interventions involved including biological agents and radiotherapy; (6) total neoadjuvant chemotherapy.

After a preliminary review, 35 abstracts were selected for full-text evaluation. After full-text review, we excluded 19 articles (Fig. 1). The 16 articles selected are shown in Table 1.

**Table 1:** Summary of included studies.

Study	Year	Source	Design	N.	Inclusion	Type	Follow-up (mo.)	Preoperative Regimen	Docetaxel total Neoadjuvant dose (mg/m²)	Lymph	pCR(%)	R0(%)	PFS 1 y. N(%)	OS 1 y. N(%)	OS 2 y. N(%)
Schulz *et al*.^22^	2015	Western Europe	Prospective	58	T3,T4 or N+	Gastric, GEJ	24	FLOT	250–300	D2	20	86	39(67.2)	46(79.3)	33(58)
Park *et al*.^11^	2013	Eastern	Prospective	41	T3,T4 or N+	Gastric, GEJ	27.2	DOS	150	D2	14.6	97.6	39(94.9)	NR	NR
Al Batran *et al*. (FLOT 3)^23^	2017	Western Europe	Prospective	51	≥T2 or N+	Gastric, GEJ	30.3	FLOT	200	D2	NR	81.6	38(75)	43(84)	35(70)
Al Batran *et al*. (FLOT 4 Phase II)^5^	2016	Western Europe	Prospective	128	≥T2 or N+	Gastric, GEJ	NE	FLOT	200	D2	16	85	NR	NR	NR
Al Batran *et al*. (FLOT 4 Phase III)^6^	2017	Western Europe	Prospective	356	≥T2 or N+	Gastric, GEJ	43	FLOT	200	D2	NR	84	241(67.6)	297 (83)	242(68)
Fonseca *et al*.^25^	2011	Western Europe	Prospective	26	T3, T4 or N+	Gastric, GEJ, DE	NR	DCX	210	NR	9	NR	NR	NR	NR
Thuss-Patience *et al*.^26^	2012	Western Europe	Prospective	51	T3, T4 or N+	Gastric, GEJ, DE	24.8	DCX	225	D2	13.7	90	41(80)	44(88)	36(70.8)
Ferri *et al*.^27^	2012	Western	Prospective	43	T1N1, ≥T2 or N+	Gastric, GEJ, DE	37	DCF	225	D2	10	100	NR	43(98)	31(74)
Hosoda *et al*.^18^	2015	Eastern	Retrospective	19	≥T1 or N+	GEJ, Siewert II	36	DCS	120	D2	NR	NR	15(78)	19(100)	16(83)
Sudarshan *et al*.^9^	2015	Western	Retrospective	86	T3, T4 or N+	GEJ, DE	40	DCF	225	D2	7	91	NR	80(93)	64(75)
Solomon *et al*.^24^	2011	Western	Prospective	29	T3 or N1	GEJ, DE	20,2	FLOD	150	NR	16,7	NR	18(62.3)	21(72.4)	12(43.7)
Favi *et al*.^19^	2017	Western Europe	Retrospective	40	≥T3	GEJ Siew. I and II	22.8	FLOT	150–300	NR	12	85	NR	29(72)	24(60)
Ito *et al*.^10^	2017	Eastern	Prospective	52	≥T2 and N+	Gastric	NR	DCS	80–120	D2	0	84.6	NR	NR	NR
Bayraktar *et al*.^20^	2012	Western	Retrospective	31	NR	Gastric, GEJ	17	DCF	225	NR	3,2	83	18(60)	25(80)	15(50)
Sun *et al*.^17^	2011	Eastern	Prospective	29	Borrmann Type IV	Gastric	NR	DCF	225	NR	6,9	51,7	NR	16(55)	9(31)
Fiteni *et al*.^21^	2016	Western Europe	Retrospective	41	≥T1 or N+	Gastric, GEJ	NR	DCF	225	NR	7	93	NR	34(83)	23(56)

mo.: months; Lymph: lymphadenectomy; pCR: pathological complete response; R0: resection rates with no gross tumor remains in the tumor bed; PFS: Progression free survival; OS: Overall Survival; N: Number; GEJ: Gastroesophageal Junction Tumors; DE: Distal Esophagus; FLOT: fluorouracil, oxaliplatin and docetaxel; DOS: docetaxel, oxaliplatin and S1; DCX: docetaxel, cisplatin and capecitabine; DCF: docetaxel, cisplatin and fluorouracil; FLOD: 5-Fluorodeoxyuridine, oxaliplatin and docetaxel; DCS: docetaxel, cisplatin and S1; NR: Not Reported; y.: year; Siew.:Siewert.

### Definitions, Outcomes of Interest, and Data Extraction

Two authors (P.L.S.U.J. and V.M.S.) extracted data of all included studies using a standardized data collection form. Primary outcomes were evaluating docetaxel based perioperative chemotherapy regimens and pathologic complete response rate (pCR), resection rates (R0) and overall survival (OS). Secondary outcomes were evaluating progression free survival (PFS), correlation with docetaxel dose delivered and toxicity of the regimens. Data of protocols were collected as published in the studies. When only survival and progression free survival curves were available, an estimation of the event rate was done by visual evaluation of the survival plots. Outcomes were extracted for groups of patients with docetaxel chemotherapy regimens. Other data collected included source of the article, Lauren histological types and lymphadenectomy performed.

### Data synthesis and analysis

We combined data using a random-effects-model meta-analysis, because we assumed that results of different studies depend not only on the investigated sample variables and the covariables but also on other factors. Fixed-effects analysis allows inference only to studies similar to those included in the meta-analysis, and random-effects analysis allows inference more broadly^13^. Overall rates were estimated with 95% confidence interval as well as effect sizes on the meta-regression models. We considered no effect when this interval contained the value 1 and the p-value for the effect was greater than 5%. The I2 index was used to measure the heterogeneity of the results of different studies. The index can vary between negative values (assumed 0%) to 100%. Higgins *et al*.^14^ suggested that up to 25% is a small degree of heterogeneity, up to 50% moderate degree and 75% or more is a high degree of heterogeneity. We also used the tau2 index and the Cochran Q test to evaluate heterogeneity.

Publication bias was evaluated using the funnel plot and analyses were performed using the R^15^ and Meta^16^ packages, considering a significance level of 5%. Because we noticed significant heterogeneity of the data, we performed a sensitivity analysis by stepwise exclusion of each study and by metaregression of docetaxel doses, in at least one of the included studies the inclusion criteria were associated with worse outcomes: patients with Bormann Type IV disease^17^.

## Results

Summarized information of each of the 16 included studies is described in Table 1. All studies were published after 2011. Sample size varied significantly, from 19 patients in Hosoda *et al*.^18^ to 356 in Al Batran *et al*.^6^. Most studies were based on prospective data, and only 5 studies evaluated retrospective data^9,18–21^. The chemotherapy regimens included are: FLOT: fluorouracil, oxaliplatin and docetaxel; DOS: docetaxel, oxaliplatin and S1; DCX: docetaxel, cisplatin and capecitabine; DCF: docetaxel, cisplatin and fluorouracil; FLOD: 5-Fluorodeoxyuridine, oxaliplatin and docetaxel; DCS: docetaxel, cisplatin and S1.

The article FLOT4 from Al Batran *et al*.^6^ is the only phase III trial evaluated; in this case the outcomes were extracted from the FLOT arm only. Five studies evaluated the FLOT regimen^5,6,19,22,23^. Only one study evaluated a regimen containing 5-Fluorodeoxyuridine^24^. Ten studies evaluated DCF and its modifications^9–11,17,18,20,21,25–27^.

There was no significant variation of enrolled patients in the studies, most of them included Gastric and Gastroesophageal Junction Tumors, five studies included distal esophagus^9,24–27^, and the majority ≥T2 or N + disease stages. The study from Sun *et al*.^17^ included Borrmann IV disease.

The neoadjuvant docetaxel dose ranged between 120–300 mg/m² and the percentages of delivery were high, between 87 to 100%. In case of postoperative chemotherapy regimen, the delivery was lower, varying between 40–80%. Additional data collected from the studies were included, as well as different regimens in the postoperative treatment that can be seen in Appendix 1.

**Figure 2:**
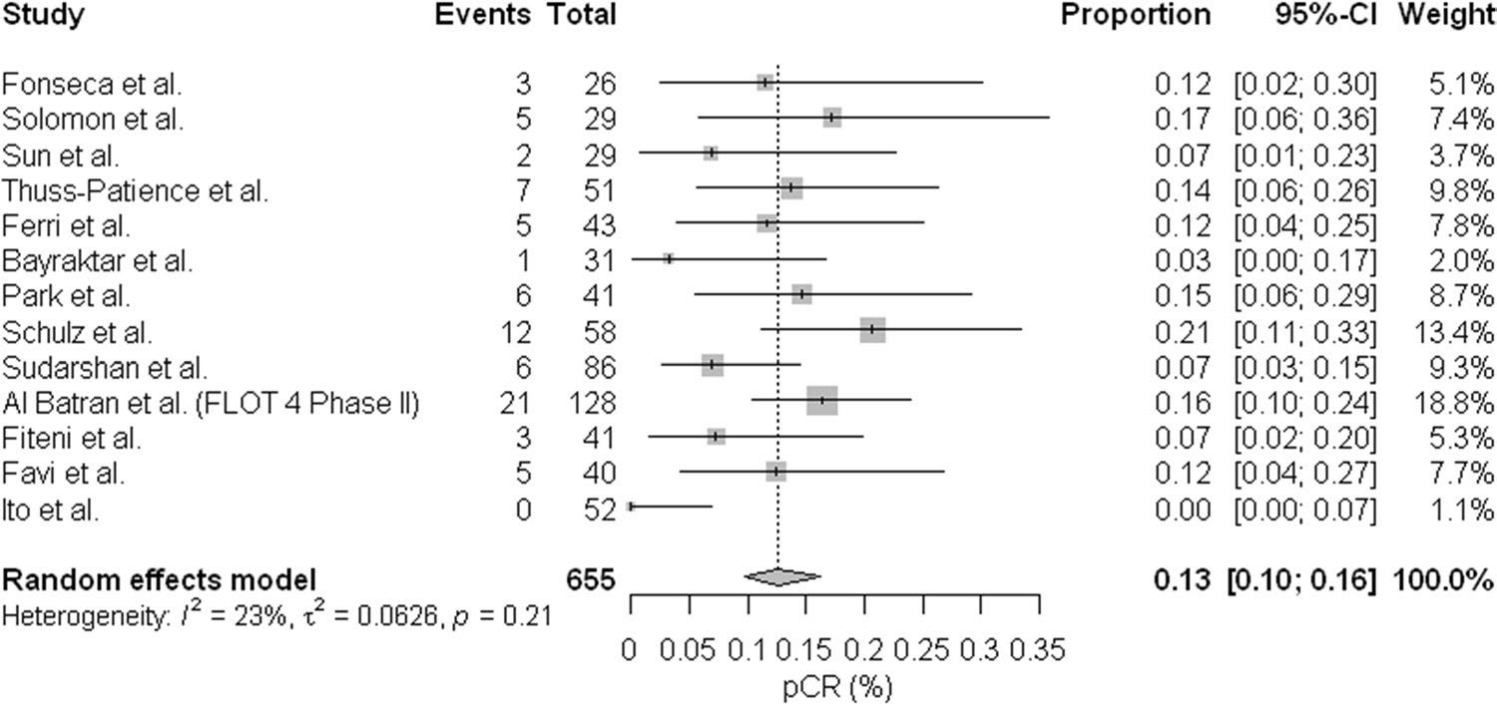
Meta-analysis of pathologic complete response. Comparing the studies with meta-analysis we can see that the regimens are similar in terms of pCR (0.13; 95% confidence interval [CI], [0.1; 0.16], without significant heterogeneity (I2 = 23%).

**Figure 3:**
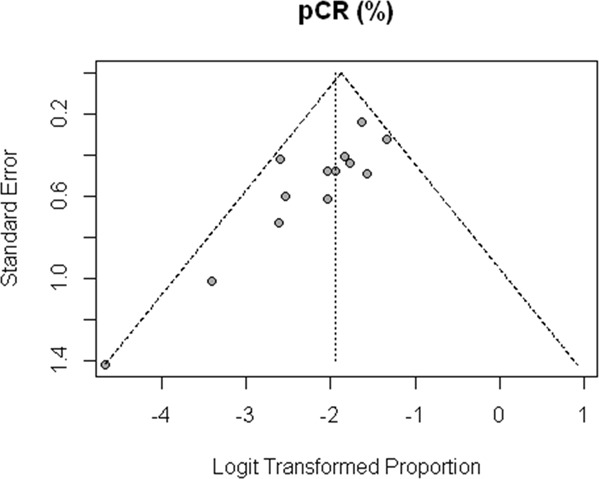
Funnel plot of pathological complete responses rates. The funnel plot shows lack of studies with complete pathological response rates higher than the polled average.

**Figure 4:**
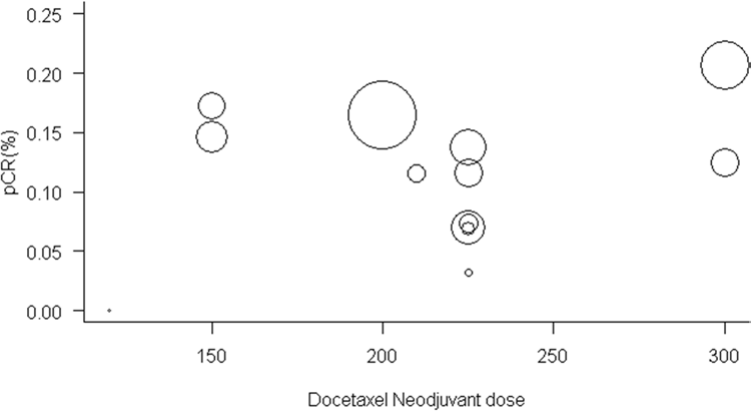
Relationship between complete pathological response rate and neoadjuvant dose of Docetaxel. The metaregression shows no association between dose and rate of complete pathological response: OR (1.20; 95% confidence interval [CI], [0.64; 2.29].

The proportion of cases with complete pathological response was available for 13 studies, the pCR ranged between 0–20%, comparing the studies with meta-analysis that we can see that the regimens are similar in terms of pCR (0.13; 95% confidence interval [CI], [0.1; 0.16] Fig. 2, without significant heterogeneity (I2 = 23%). Regarding the publication bias, the funnel plot (Fig. 3) shows a lack of studies with complete pathological response rates higher than the polled average. However, those studies were predicted to have wider standard errors suggesting that they were small and their effects in the analysis were low. We further performed a metaregression of the total doses of docetaxel conducted in the preoperative setting; we did not find association between dose and rate of complete pathological response: OR (1.20; 95% confidence interval [CI], [0.64; 2.29] (Fig. 4). In the metaregression model according to the chemotherapy regimens used, the model suggests that the FLOT regimen presents a greater chance of pCR than the other regimens. We also evaluated the impact of D2 lymphadenectomy on these results and there was no statistical association (Appendix 2).

**Figure 5:**
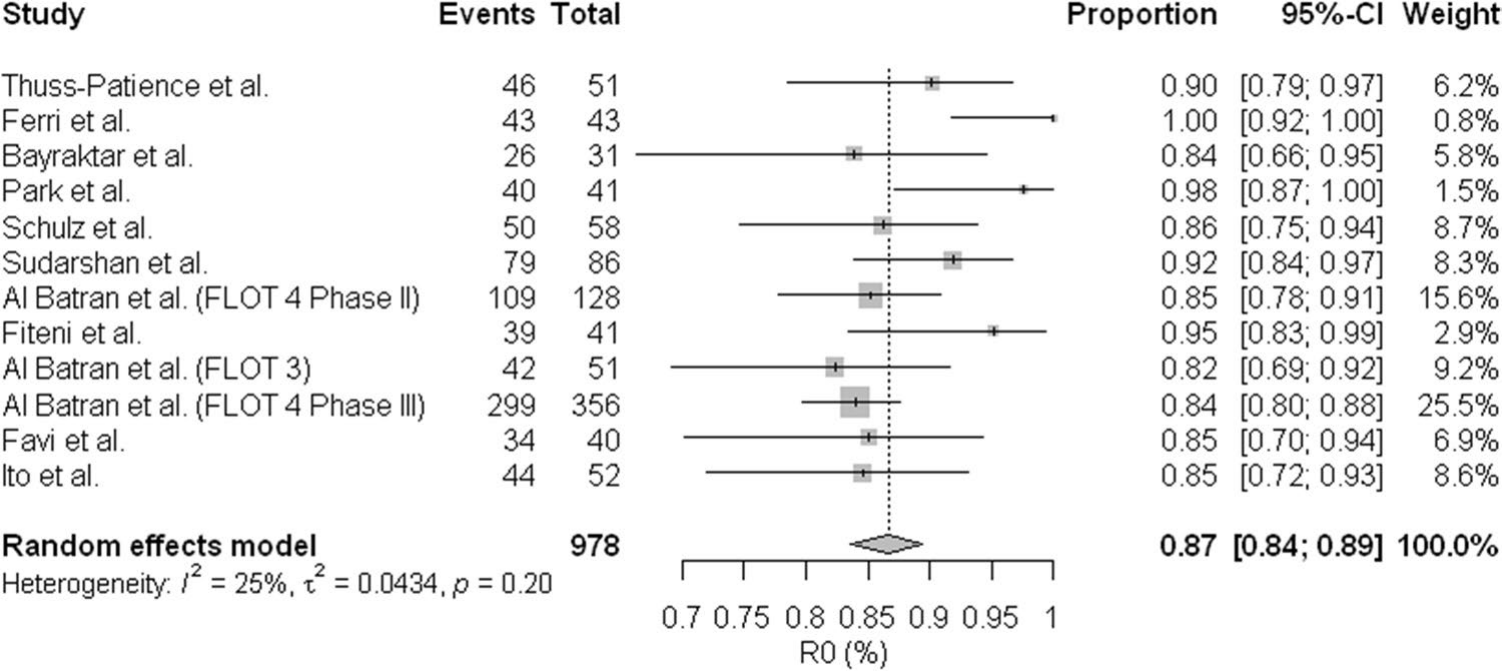
Meta-analysis for R0 (Excluded Sun *et al*.^15^). The R0 rate is similar between all studies: R0 (0.87; 95% confidence interval [CI], [0.84; 0.89]; and low heterogeneity (I² = 25%).

**Figure 6:**
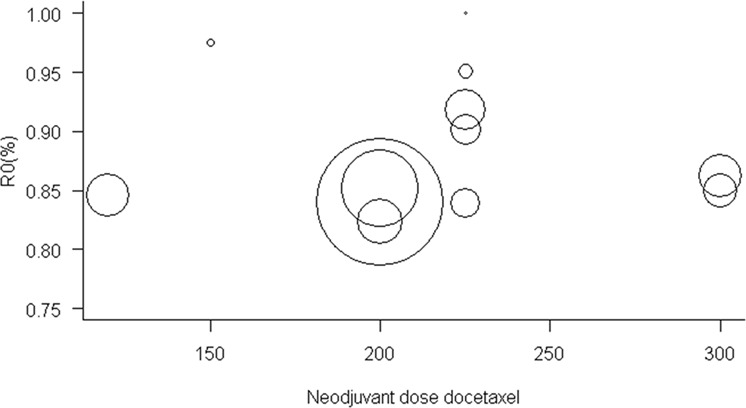
Relationship between R0 and neoadjuvant dose of Docetaxel (Excluding Sun *et al*.^15^). No association was found between dose of docetaxel and R0 rates: OR (1.00; 95% confidence interval [CI], [0.99; 1.00].

The proportion of cases with R0 resection rate was available for 13 of the 16 studies, and the values observed were between 51.7% and 100.0%. In the evaluation of meta-analysis model, we can observe a high rate of heterogeneity of these results (I² = 66%) Appendix 3, apparently related to the extremely low R0 rate (51.7%) of Sun *et al*. study.^17^, mainly due to the fact that the study contemplates only patients with clinical diagnosis Bormann IV and almost 30% of the patients were submitted to palliative resections in the perioperative chemotherapy regimen. When conducting a new meta-analysis for sensitivity analysis with the withdrawal of this study, we observed that the R0 rate is similar between all studies: R0 (0.87; 95% confidence interval [CI], [0.84; 0.89]; and low heterogeneity (I² = 25%) Fig. 5, suggesting that the regimens are similar in this evaluation. We also did not find association between dose of docetaxel and R0 rates: OR (1.00; 95% confidence interval [CI], [0.99; 1.00] (Fig. 6). Regarding the publication bias, the funnel plot shows lack of studies with R0 rates lower than the polled average. Similarly, in the analysis of pCR, those studies were predicted to have wider standard errors, suggesting that they were small and their effects in the analysis would be low (Appendix 4).

**Figure 7:**
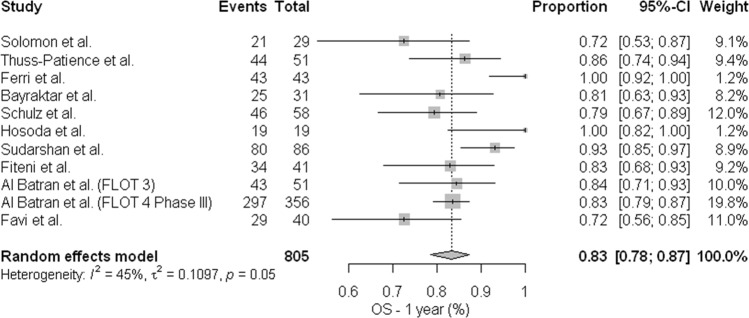
Meta-analysis for overall survival after one year (Excluding Sun *et al*.^15^). Overall Survival rate is similar between all studies: OS (0.83; 95% confidence interval [CI], [0.78; 0.87].

We were able to obtain information about overall survival in 12 studies, the number of survivors after one year ranged from 55% to 100%. Based on previous results that included the study from Sun *et al*.^17^, with worst outcomes compared with the other studies, we performed meta-analysis of overall survival with and without Sun *et al*.’s study. Again, we can note the high rate of heterogeneity of the results (I² = 65%) Appendix 5, apparently related to the extremely low survival rates after one year (55%) in Sun *et al*.’s study.^17^, particularly related to worse prognosis and surgical palliative treatment in cases of Borrmann IV disease. When a new meta-analysis for sensitivity analysis with the withdrawal of this study was conducted, we noted that the OS rate was similar between all studies: OS (0.83; 95% confidence interval [CI], [0.78; 0.87]; heterogeneity (I² = 45%) (Fig. 7). For metaregression, we used total dose of docetaxel, including postoperative treatment, and we did not find association between dose of perioperative docetaxel and OS rates: OR (1.00; 95% confidence interval [CI], [0.99; 1.00]) (Appendix 6). In our analysis D2 lymphadenectomy had a positive effect in overall survival: OR 0.42 (95% confidence interval [CI], [0.22; 0.80] p 0.008). Regarding the publication bias, the funnel shows similar results to the previous analysis of pCR and R0 rates, with a lack of small studies with wider standard errors (Appendix 7).

The progression free survival rate at the end of one year was recorded for only eight studies and ranged from 60% to 94.9%. In this analysis we also found similar results of PFS (0.72; 95% confidence interval [CI], [0.64; 0.78]); with moderate heterogeneity (I² = 57%) (Appendix 8). Our analysis showed no association between dose of perioperative docetaxel and PFS rates: OR (0.78; 95% confidence interval [CI], [0.51; 1.19]), neither on D2 lymphadenectomy: OR (0.50; 95% confidence interval [CI], [0.22; 1.12]). However, this interpretation is limited due to the small number of trials included.

Some studies provided data on overall survival at 2 and 3 years, the analysis of these data were performed but there is a high rate of heterogeneity, probably related to the effects of chemotherapy regimens performed during metastatic disease. Unfortunately, the regimens used could not be accessed to evaluate their impact on these results (Appendix 9, 10).

Regarding the toxicity of the regimens, we found that docetaxel based perioperative regimens are quite toxic, the FLOT combination had higher levels of neurosensory toxicity and mucositis grade 3 and 4 compared with other regimens, the DCF modifications with infusional fluorouracil seems to be better tolerated than the regimens with oral fluoropyrimidines mainly to hematological toxicity, others differences presumably are intensified by the use of colony stimulating factors only in character of secondary prophylaxis Table 2, Appendix 1.

**Table 2:** Description of observed adverse GRADE 3–4 event rates.

	FLOT	mDCF	DCX/DCS/DOS	FLOD
Leukopenia	21–28%	Not reported	**19**–**49%**	Not reported
Neutropenia	29–52%	6–13%	**27**–**76%**	27%
Neutropenic Fever	5%	5.7–13%	**2.3**–**21%**	10%
Thrombocytopenia	0–1.7%	0%	**2**–**17.1%**	3.4%
Diarrhea	7–12%	0–10%	2–19%	**17.2%**
Mucositis	**2**–**7%**	6%	0–4%	Not reported
Neurosensory	**5% -12%**	0.0%	0%	Not reported
Mortality	**1**–**3.4%**	0–3%	0–2%	**6.8%**

FLOT: fluorouracil, oxaliplatin and docetaxel; DOS: docetaxel, oxaliplatin and S1; DCX: docetaxel, cisplatin and capecitabine; DCF: docetaxel, cisplatin and fluorouracil; FLOD: 5-Fluorodeoxyuridine, oxaliplatin and docetaxel; DCS: docetaxel, cisplatin and S1.

## Discussion

This meta-analysis of observational and prospective studies with a combined sample size of 1,081 patients suggests that perioperative docetaxel regimens in gastric and esophagogastric junction tumors are equally effective in all outcomes evaluated, including rates of pathological complete response, resection rates (R0), progression free survival and overall survival.

After publication of the phase 3 trial comparing FLOT and ECF^6^ regimens that demonstrated benefit in all outcomes evaluated including overall response rate and survival, the FLOT regimen became accepted worldwide as the standard perioperative treatment for gastric and esophagogastric junction tumors. However, several authors have questioned whether the FLOT regimen should be considered a standard because of concerns on safety and efficacy compared against better tolerated regimens without docetaxel such as FOLFOX (infusional fluorouracil plus oxaliplatin), especially for elderly and fragile patients^28,29^. The DCF regimen had previously demonstrated to be superior to the two-drug regimens, but in a metastatic disease scenario, in V325 study^30^. Regimen modifications concerning tolerability and toxicity issues were extensively studied with apparently similar results although there is no direct comparison between them^9–11,17,18,20,21,25–27^. When analyzing the rates of complete pathological response in the various docetaxel regimens including FLOT and modified DCF regimens, we can see that there is great similarity between the regimens and their efficacy (0.13; 95% confidence interval [CI], [0.1; 0.16]) heterogeneity (I2 = 23%).

A concerning issue of perioperative treatment, the R0 resectability rate, a concept defined as absence of tumor in the tumor bed, with complete resection of the lesion, was also evaluated and demonstrated that rates were similar in the meta-analysis (0.87; 95% confidence interval [CI], [0.84; 0.89]; Heterogeneity (I ² = 25%). This finding was refined after evaluation of sensitivity resulting from the exclusion of the study by Sun *et al*.^17^. This analysis made the data more homogeneous. Although the study by Sun *et al*.^17^ was included in the (pCR) assessments, their study clearly indicated that it was the main responsible for the heterogeneity of the analysis in resectability rate, which was confirmed by withdrawing of the study. The inferior results of resectability of this study when compared with other studies included derived from the reserved prognosis of the inclusion condition Borrmann IV^31–33^.

The overall survival assessment also showed that the regimens are equally effective in 1-year survival, (0.83; 95% confidence interval [CI], [0.78; 0.87]; heterogeneity (I ² = 45%), also with acceptable heterogeneity after the exclusion of the Sun *et al*. study^17^. The data of overall survival in 2 and 3 years were also obtained in the studies, but in the analysis of these outcomes, it is observed high heterogeneity of results probably due to survival after disease recurrence which is strongly influenced by systemic treatment in metastatic lines. Moreover, the information of treatment regimens used after recurrence is not present in most studies making analysis of sensitivity or metaregression impossible to be performed. A factor that showed impact was the D2 lymphadenectomy rate, with results of better survival when performed (OR 0.42 (95% confidence interval [CI], [0.22; 0.80] P 0.008). The surgery with D2 lymphadenectomy is considered a standard treatment in several centers around the world due to greater lymph node sampling and eradication of loco regional disease^34,35^.

When carefully examined the studies for progression-free survival analysis, a study stood out in terms of PFS in 1 year. The study by Park *et al*.^11^ presented high levels of progression-free survival (94.9%), and probably became the source of heterogeneity of this analysis, it is worth mentioning a particularity of it, in adjuvant treatment to patients who received S1 for 1 year, what is approved for adjuvant setting based on a positive randomized phase 3 Eastern study^36^. This treatment for 1 year and the totality of patients being operated with D2 Lymphadenectomy may have influenced the control in recurrences more effectively in the first year. These results of our study compared with others in terms of recurrence-free survival in the first year may be hypotheses generator for future randomized studies of perioperative regimens.

When comparing the toxicity of the regimens, we observed that the docetaxel-based regimens present relevant toxicities despite being manageable. As it is expected, regimens that contemplate oxaliplatin present higher rates of neurosensory toxicities, sometimes irreversible, and platinum-based treatments frequently contemplate hematological toxicities. Regarding the use of fluoropyrimidines apparently the use of oral fluoropyrimidines is more toxic, including capecitabine and S1, a possible cause may be related to increased exposure time to the chemotherapeutic agent when compared to periodic infusions, differences related to frequency of laboratories in the trials and lower tolerability in Caucasian patients^25,26,37,38^. Our analysis raised the studies that used regimens with infusional fluorouracil, they were better tolerated, in addition, infused fluorouracil in more than 24 hours apparently is also better tolerated. The FLOT regimen presents fluorouracil infusion at 24 hours. It is noteworthy that a large part of the studies did not adequately report or omitted the toxicities of the regimens used, especially in the DCF studies and their modifications^9,17,20,21,27^.

The analysis of chemotherapy and docetaxel regimens was performed in order to evaluate the best regimen and if the dose of docetaxel was influential in the results obtained. In terms of efficacy only the FLOT regimen in the PCR analysis presented possibly better results, in other outcomes there was no statistical significance of any other regimens. In concerning to the effect of oxaliplatin, cisplatin, fluorouracil and oral fluoropyrimidines we assumed that the efficacy of oxaliplatin when compared to cisplatin and infusional fluorouracil when compared to oral fluoropyrimidines are equivalent in gastric and esophagogastric junction tumors. In fact, this analysis has been performed in a four arms randomized phase 3 study including more than 800 patients with metastatic disease; the study evaluated the interpolation of oxaliplatin with cisplatin and fluorouracil with capecitabine in regimens of 3 drugs based on epirubicin and demonstrated that these drugs have the same efficacy^39^; even a meta-analysis compared the fluoropyrimidines, infusional and oral, and also reached similar results^40^. As the difference between the FLOT regimen and the ECF regimen is supposed to be the addition of taxane, our analysis aimed to evaluate different doses of this drug in terms of toxicity and efficacy. The epirubicin in several studies showed no additional benefits in the combinations, both in the scenario of localized disease and in metastatic disease^41,42^.

This meta-analysis presents some limitations; data heterogeneity, publication bias and lack of patient-level data. Due to the fact it contemplates only data published or presented in Congress Annals, we clearly notice in the analysis of funnel plot the lack of studies with a small number of patients, therefore, leading to results inferior to the pattern found. This publication bias is intrinsic to this type of study, but it becomes less relevant because such studies were predicted to have wider standard errors, which suggested that their effects in the analysis would be low. To reduce the heterogeneity of our data, a new sensitivity analysis was adequate with the withdrawal of the study by Sun *et al*.^17^, which was the only study that included Bormann IV patients, and presented a more reliable result.

Finally, individual patient data could have helped to identify other sources of heterogeneity, particularly in analysis of overall survival in 2 or 3 years. The strength of our analysis is related to the inclusion of a good number of studies with a combined sample size of more than 1,000 patients and consistent findings.

## Conclusion

The results of the present analysis suggest that docetaxel perioperative based regimens are equivalent in terms of pCR, resection rates (R0) and overall survival at least in the first year. These results could provide evidence to evaluate modified docetaxel regimens, particularly modified DCF or even FLOT regimens with lower doses of docetaxel, evaluating similar and less toxic treatments with the same effectiveness, thus increasing the spectrum of patients who will benefit from this strategy.

## Supplementary information

Supplementary dataset
