# Supplementary material for: Systematic review and meta-analysis of docetaxel perioperative chemotherapy regimens in gastric and esophagogastric tumors
Source: Sci Rep. 2019 Nov 1;9:15806. doi: 10.1038/s41598-019-52334-y (PMC6825156; doi:10.1038/s41598-019-52334-y)
Supplement: Supplementary file 1 — Supplementary dataset [file 41598_2019_52334_MOESM1_ESM.pdf]

**Systematic review and meta-analysis of docetaxel perioperative chemotherapy regimens in gastric and esophagogastric tumors**

Pedro Luiz Serrano Usón Junior, MD
Vanessa Montes Santos, MD
Diogo Diniz Gomes Bugano, MD
Elivane da Silva Victor
Edna Terezinha Rother
Fernando Cotait Maluf, MD, PhD.

## Appendix 1: Full Summary of included studies

| Study | Year | Source | Design | Diffuse (n) | Intestinal (n) | Mixed (n) | Inclusion |
|---|---|---|---|---|---|---|---|
| Schulz et al. | 2015 | Western Europe | Prospective | 15 | 36 | 4 | T3,T4 or N+ |
| Park et al. | 2013 | Eastern | Prospective | 27 | 14 | 0 | T3,T4 or N+ |
| Al Batran et al. (FLOT 3) | 2017 | Western Europe | Prospective | 20 | 17 | 9 | ≥T2 or N+ |
| Al Batran et al. (FLOT 4 Phase II) | 2016 | Western Europe | Prospective | 34 | 52 | 11 | ≥T2 or N+ |
| Al Batran et al. (FLOT 4 Phase III) | 2017 | Western Europe | Prospective | 95 | 159 | NE | ≥T2 or N+ |
| Fonseca et al. | 2011 | Western Europe | Prospective | NR | NR | NR | T3, T4 or N+ |
| Thuss-Patience et al. | 2012 | Western Europe | Prospective | 11 | 20 | NR | T3, T4 or N+ |
| Ferri et al. | 2012 | Western | Prospective | NR | NR | NR | T1N1, ≥T2 or N+ |
| Hosoda et al. | 2015 | Eastern | Retrospective | NR | NR | NR | ≥T1 or N+ |
| Sudarshan et al. | 2015 | Western | Retrospective | NR | NR | NR | T3, T4 or N+ |
| Solomon et al. | 2011 | Western | Prospective | NR | NR | NR | T3 or N1 |
| Favi et al. | 2017 | Western Europe | Retrospective | NR | NR | NR | ≥T3 |
| Ito et al. | 2017 | Eastern | Prospective | 24 | 29 | 0 | ≥T2 and N+ |
| Bayraktar et al. | 2012 | Western | Retrospective | NR | NR | NR | NR |
| Sun et al. | 2011 | Eastern | Prospective | NR | NR | NR | Borrmann Type IV |
| Fiteni et al. | 2016 | Western Europe | Retrospective | NR | NR | NR | ≥T1 or N+ |

**Legend: NR: not reported**

**Appendix 2: Results of the meta-regression models applied to pCR data**

| pCR | Odds ratio | CI 95% | p-value | $I^2$ | p-value Q |
|---|---|---|---|---|---|
| Regimen-Preoperative | | | | | |
| DCF | 1 (Ref.) | | | 0.0% | 0.880 |
| DCS | 0.113 | (0.007; 1.913) | 0.131 | | |
| DCX | 1.777 | (0.775; 4.073) | 0.174 | | |
| DOS | 2.034 | (0.750; 5.519) | 0.163 | | |
| FLOD | 2.472 | (0.836; 7.307) | 0.102 | | |
| FLOT | 2.429 | (1.324; 4.456) | 0.004 | | |
| Docetaxel total Neoadjuvant dose (x100mg/m²) | 1.209 | (0.644; 2.293) | 0.549 | 27.1% | 0.178 |
| Lymphadenectomy D2 | 0.738 | (0.392; 1.387) | 0.345 | 22.4% | 0.223 |

**Legend:** pCR: pathological complete response; FLOT: fluorouracil, oxaliplatin and docetaxel; DOS: docetaxel, oxaliplatin and S1; DCX: docetaxel, cisplatin and capecitabine; DCF: docetaxel, cisplatin and fluorouracil; FLOD: 5-Fluorodeoxyuridine, oxaliplatin and docetaxel; DCS: docetaxel, cisplatin and S1;

## Appendix 3:  Forest plot for R0 rates

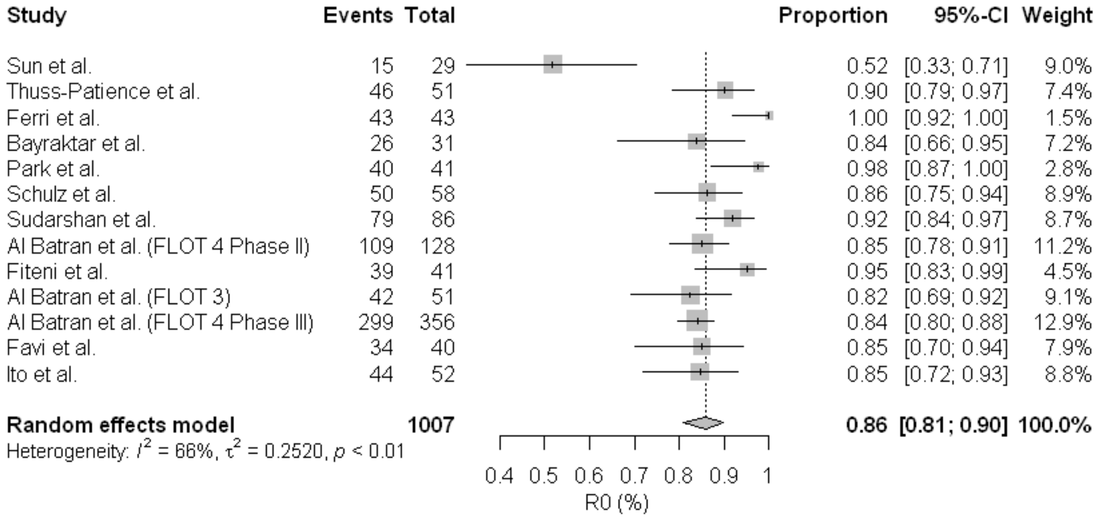

**Appendix 4: Funnel plot for R0 rates (Excluding Sun et al. [16])**

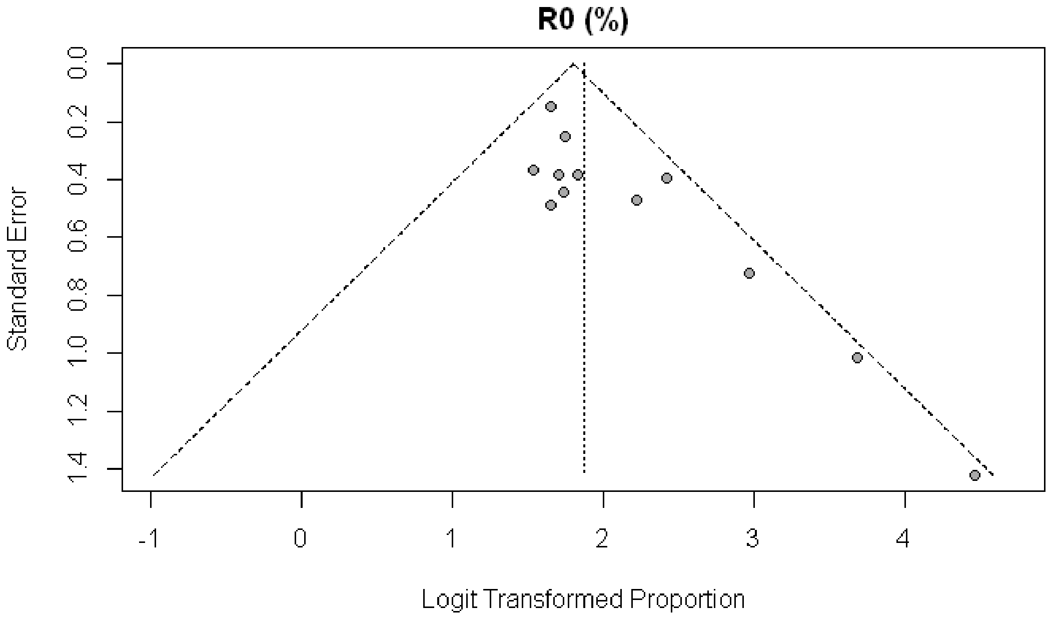

## Appendix 5: Forest plot for overall survival in one year

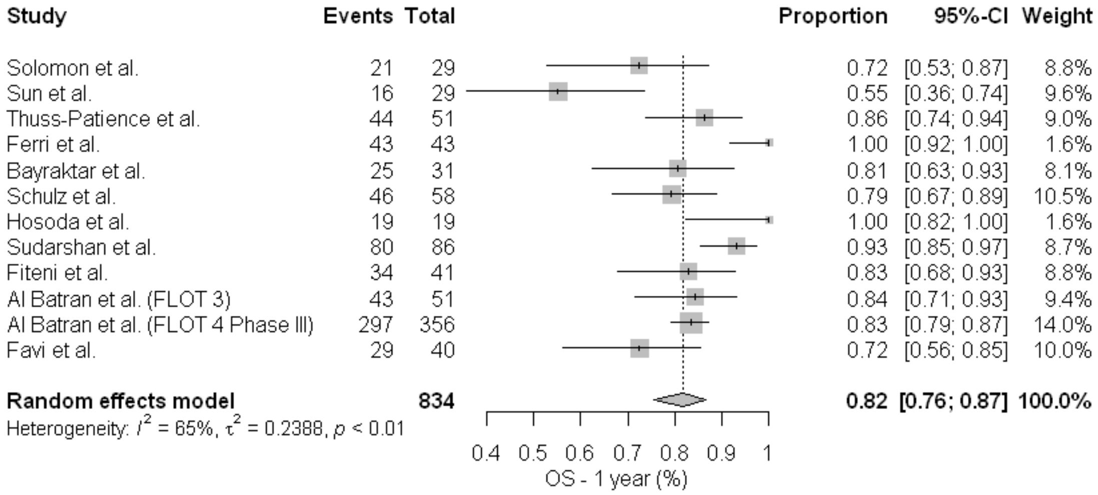

**Appendix 6: Relation between overall survival after one year and total dose of Docetaxel (Excluding Sun et al. [15])**

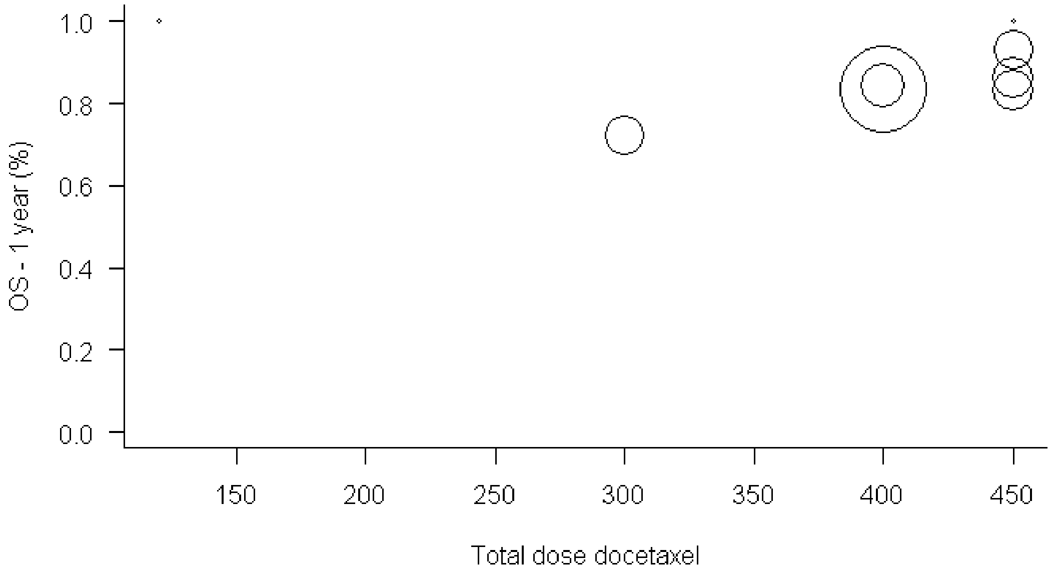

**Appendix 7: Funnel plot for overall survival at one year (Excluding Sun et al. [15])**

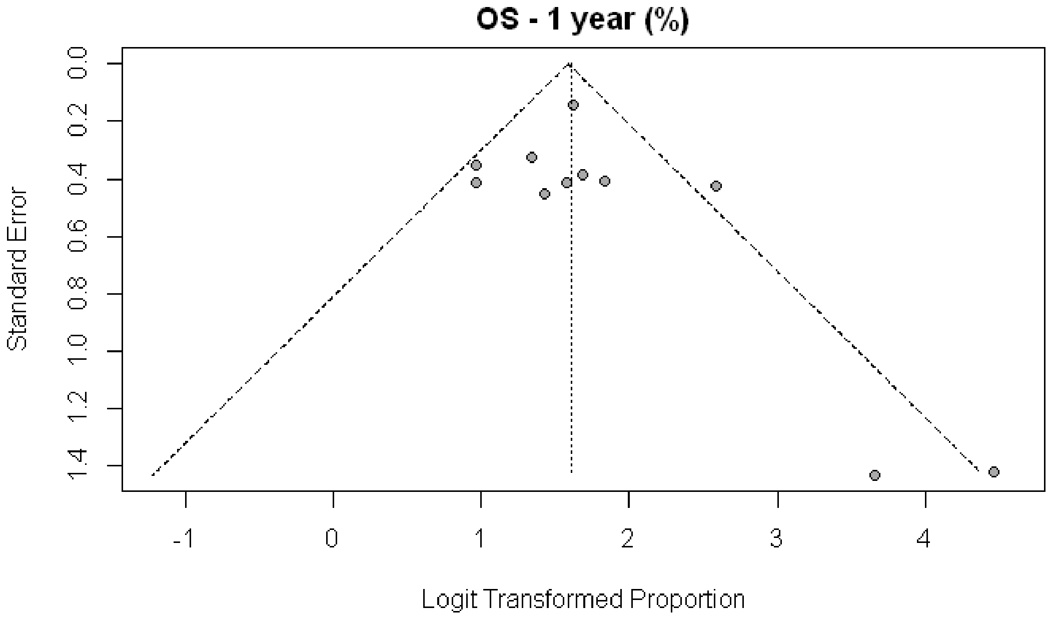

## Appendix 8: Forest plot for progression free survival in one year

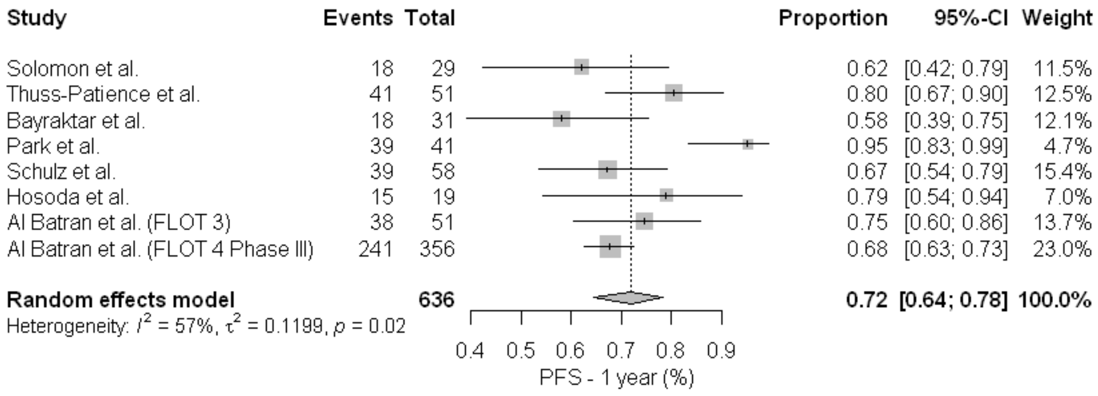

# Appendix 9: Forest plot for overall survival in two years

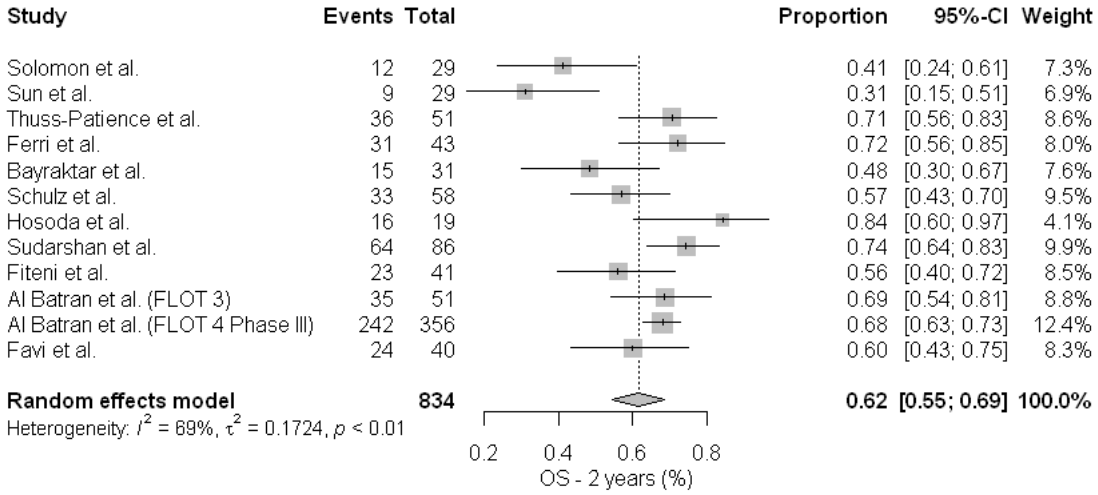

## Appendix 10: Forest plot for overall survival in three years

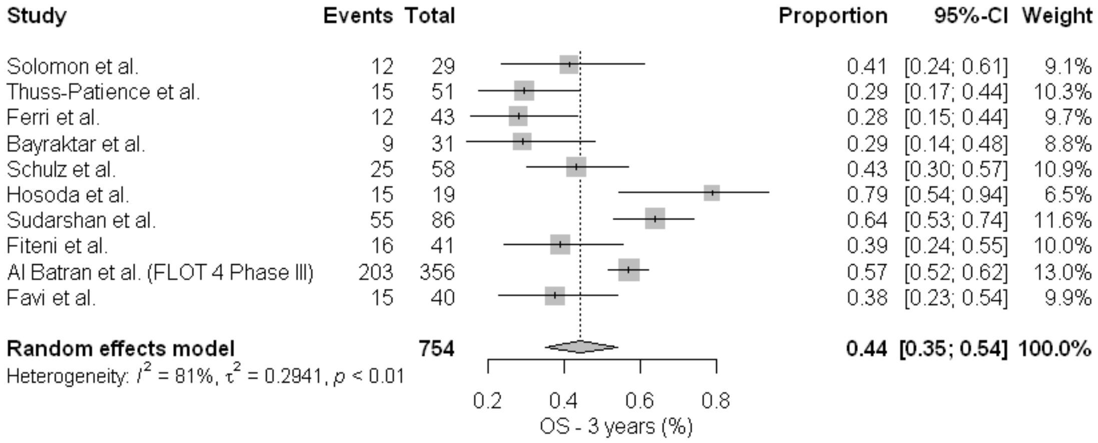

| Section/topic | # | Checklist item | Reported on page # |
|---|---|---|---|
| **TITLE** | | | |
| Title | 1 | Identify the report as a systematic review, meta-analysis, or both. | 1 |
| **ABSTRACT** | | | |
| Structured summary | 2 | Provide a structured summary including, as applicable: background; objectives; data sources; study eligibility criteria, participants, and interventions; study appraisal and synthesis methods; results; limitations; conclusions and implications of key findings; systematic review registration number. | Abstract structured as journal requires |
| **INTRODUCTION** | | | |
| Rationale | 3 | Describe the rationale for the review in the context of what is already known. | 3 |
| Objectives | 4 | Provide an explicit statement of questions being addressed with reference to participants, interventions, comparisons, outcomes, and study design (PICOS). | 4,5 |
| **METHODS** | | | |
| Protocol and registration | 5 | Indicate if a review protocol exists, if and where it can be accessed (e.g., Web address), and, if available, provide registration information including registration number. | 4 |
| Eligibility criteria | 6 | Specify study characteristics (e.g., PICOS, length of follow-up) and report characteristics (e.g., years considered, language, publication status) used as criteria for eligibility, giving rationale. | 4 |
| Information sources | 7 | Describe all information sources (e.g., databases with dates of coverage, contact with study authors to identify additional studies) in the search and date last searched. | 4 |
| Search | 8 | Present full electronic search strategy for at least one database, including any limits used, such that it could be repeated. | 4 |
| Study selection | 9 | State the process for selecting studies (i.e., screening, eligibility, included in systematic review, and, if applicable, included in the meta-analysis). | 4,5 |

| | | | |
|---|---|---|---|
| Data collection process | 10 | Describe method of data extraction from reports (e.g., piloted forms, independently, in duplicate) and any processes for obtaining and confirming data from investigators. | 4,5 |
| Data items | 11 | List and define all variables for which data were sought (e.g., PICOS, funding sources) and any assumptions and simplifications made. | 4,5 |
| Risk of bias in individual studies | 12 | Describe methods used for assessing risk of bias of individual studies (including specification of whether this was done at the study or outcome level), and how this information is to be used in any data synthesis. | 6 |
| Summary measures | 13 | State the principal summary measures (e.g., risk ratio, difference in means). | 5,6 |
| Synthesis of results | 14 | Describe the methods of handling data and combining results of studies, if done, including measures of consistency (e.g., $I^2$) for each meta-analysis. | 5,6 |
